# The relationship between nest location selection of Barn swallows (*Hirundo rustica*) and human activity and residence

**DOI:** 10.1038/s41598-023-50149-6

**Published:** 2023-12-27

**Authors:** Minyoung Kim, Ok-Sik Chung, Jong Koo Lee

**Affiliations:** 1 Division of Life Science, Incheon National University, 119 Academy-Ro, Yeonsu-Gu, Incheon, 22012 South Korea; 2 Space and Environment Laboratory, Chungnam Institute, 73-26 Institute Road, Gongju, South Chungcheong Province 32589 South Korea

**Keywords:** Ecology, Zoology

## Abstract

We found that barn swallow (*Hirundo rustica*) breeding occurs within close proximity to humans. An evaluation of barn swallow breeding frequency and the breeding success rate of swallows at research sites, which were buildings inhabited by humans and buildings where humans had previously resided, was conducted in order to establish a relationship between the location of barn swallow nests and human habitation and activity frequency. The results demonstrated that barn swallows often breed in human-inhabited buildings. No significant relationship was observed between the wall material and the direction of the wall and the type of building, whereas a much higher proportion of the nests were located near doors with a high level of human movement. In addition, no significant correlation was observed between the location of the nest and the distance from potential resources (food, water etc.), however, a statistically significant relationship was observed between the frequency of human activity measured through the video camera and the number of nests located at a certain distance. The average number of offspring and the reproductive success rate were higher in nests located within close proximity to human activity compared to nests not located within close proximity to human activity, suggesting that the presence of humans had a positive effect on reproduction. This study show that barn swallow nesting occurs in locations where there is a human influence and humans provide implicit protection of swallows from predation, which has a significant impact on breeding.

## Introduction

The breeding process of birds is dangerous, for both the offspring and the parents; birds exhibit sensitivity to conditions such as breeding season and nest site. In particular, there is a close relation between the location of the nest chosen by the parents and the success of breeding^1^, and physical environmental factors^2^, surrounding species, nest predation^3,4^, and distance from food sources are important factors determining reproductive success^5–7^. Consequently, birds have evolved for habitat selection where factors affecting reproductive success can be satisfied^8^ and for selection of various nesting locations such as cavity nest, shrub layer, water surface, and cliffs in order to suit the breeding ecology.

In general, while for many birds, selection of nesting locations is based on avoiding interference or influence of interspecific or conspecific species, barn swallows tend to build their nests within close proximity to human living spaces. Barn swallows, which inhabit locations worldwide, including Eurasia, America, and Africa, build their nests on the walls of doors, houses, barns, and warehouses, which are man-made structures, not natural elements such as trees or shrubs^9,10^. Barn swallows are homing birds^11,12^ approximately 40% of the population returns to their previous breeding grounds. They are friendly to human, so appearing frequently in historical events and traditional fairy tales in many countries. In medieval religions, swallows are sometimes regarded as being close to the gods, and in Europe, swallows are known as spring's herald^13^. They can also be helpful in farming by consuming pests and their species is so close to that of humans that a poem was written about swallows. In Korean legend, there is also a fairy tale about a swallow who gave a seed that will produce treasure to a good farmer who healed his injured leg.

Some bird species share a habitat with humans. By living close to humans, they gain a reproductive advantage. Humans have been farming for a long time; thus, sparrows, who eat mainly grains, are aided by the supply of food around humans. The great tit (*Parus major*) is a cavity nester, breeding in holes pre-made by other species and artificial nest boxes near locations of human habitation. The domestic pigeon, a representative species, has adapted to urban areas that are densely populated by humans. They can be observed breeding in various places such as crevices in buildings and under bridges. They do not share a habitat with humans, however, there are cases involving interaction with humans. In the case of the Greater honeyguide (*Indicator indicator*), honey in the hive is a staple food, however, they are not able to consume the honey from the hive themselves, therefore, they lure humans to dig out the hive, and then eat the remaining honey from the hive. The question of whether the barn swallow chooses to nest closest to humans because it can obtain any advantage associated with humans should be considered.

The barn swallow can build nests in various places, such as bridges and cliffs, as opposed to a species that only cavity nests, like the great tit. Nevertheless, considering the reasons why swallows choose to breed in close proximity to humans in human settlements and what the benefits might be, one possibility could be nest predation. Nest predation is the most common reason for reproductive failure in birds^14,15^, and in order to avoid this, birds that are prey in the natural environment may select a breeding site located near a higher-level predator, a protective species, with the expectation that they will be protected from predators that pose a potential threat to their reproduction^16,17^. In this way, it can be supposed that swallows utilize humans as a pattern of adaptation, recognizing humans as higher-level predators than their predators and reducing the rate of predation from predators that pose a threat to their reproduction.

In this study, a comparison of the characteristics of the nest location chosen by swallows as a breeding site and the reproductive ecology such as the success or failure of breeding was performed in order to examine the causes of barn swallow breeding around humans. In particular, evaluation of various factors, including direction, location, material, and distance from the nesting wall, and food source, was performed for analysis of the relationship between humans, such as human residence in the nesting place and human activity in the nesting place. In addition, an attempt was made to understand the reasons for inhabiting areas near humans through assessment of breeding results such as changes in breeding time and success rate in order to clarify the meaning of nest location selection.

## Methods

### Study site and process

Field work was conducted in Shinan-gun, Jeollanam-do, Korea in 2020 and 2021. We selected 14 study sites on three islands, Amtae-do (34°50′38"N 126°05′32"E), Palgeum-do (34°47′10"N 126°08′11"E), and Anjwa-do (34°44′53"N 126°07′51"E), based on an appropriate ratio of vacant houses to occupied ones, and places where the population of arriving swallows is high. These sites are located in areas with many detached houses that provide easy nesting for barn swallows. In these areas there is appropriate distribution of buildings where barn swallows live and empty buildings that are no longer inhabited, so that we can observe the correlation between human habitation and activity and the barn swallow's nest selection. In addition, swallows can acquire food resources in various locations around the village, such as forests, rice fields, fields, and reservoirs, so that many barn swallows come and breed, thus this area is suitable for the purpose of this study. In the study site survey, after assigning numbers to the buildings in the area and nests located in each building, the breeding status was confirmed. The type of building where the nest was located, the material of the wall that holds the nest, human residence in the building, and the location of the nest in the building were examined, and the breeding ecology, including the number of eggs, the number of nestlings, the success rate of breeding, and the first laying date were recorded. We used the data of nesting only in the first breeding attempt. In order to understand the relationship between the frequency of human activity and the swallow’s nest position, a video camera (Xiaomi Mi 4 k) was installed in 27 random locations prior to the arrival of barn swallows and the start of nest building. Measurements of the frequency of human movement were taken for 2 h from 11:00 am to 1:00 pm in each region. In addition, the number of nests that swallows attempted to breed and whether breeding was attempted were made in the vicinity within camera angle were examined. It was not possible to record data blind because our study involved focal animals in the field.

### Statistical analysis

In this study, Frequency analysis, Simple Regression analysis, Poisson Regression analysis, and Kruskal Wallis test, which were used for data analysis, were performed using IBM SPSS Statistics 25. Specifically, a frequency analysis was performed for analysis of differences in the barn swallow's preference for breeding site selection (human residence, reuse, building type, material, and location of the nesting location, etc.). Poisson regression analysis was performed for examination of the relationship between the frequency of human movement recorded by the camera and the number of nests according to location, and a comparison of the first laying date, the wall material and building type where the swallow's nest was located, and the breeding success rate for each nest was evaluated using a nonparametric method (Kruskal–Wallis test) and post-hoc (Bonferroni) to confirm the difference between groups.

## Results

### The relationship between human habitation and the barn swallow's nest

**Figure 1:**
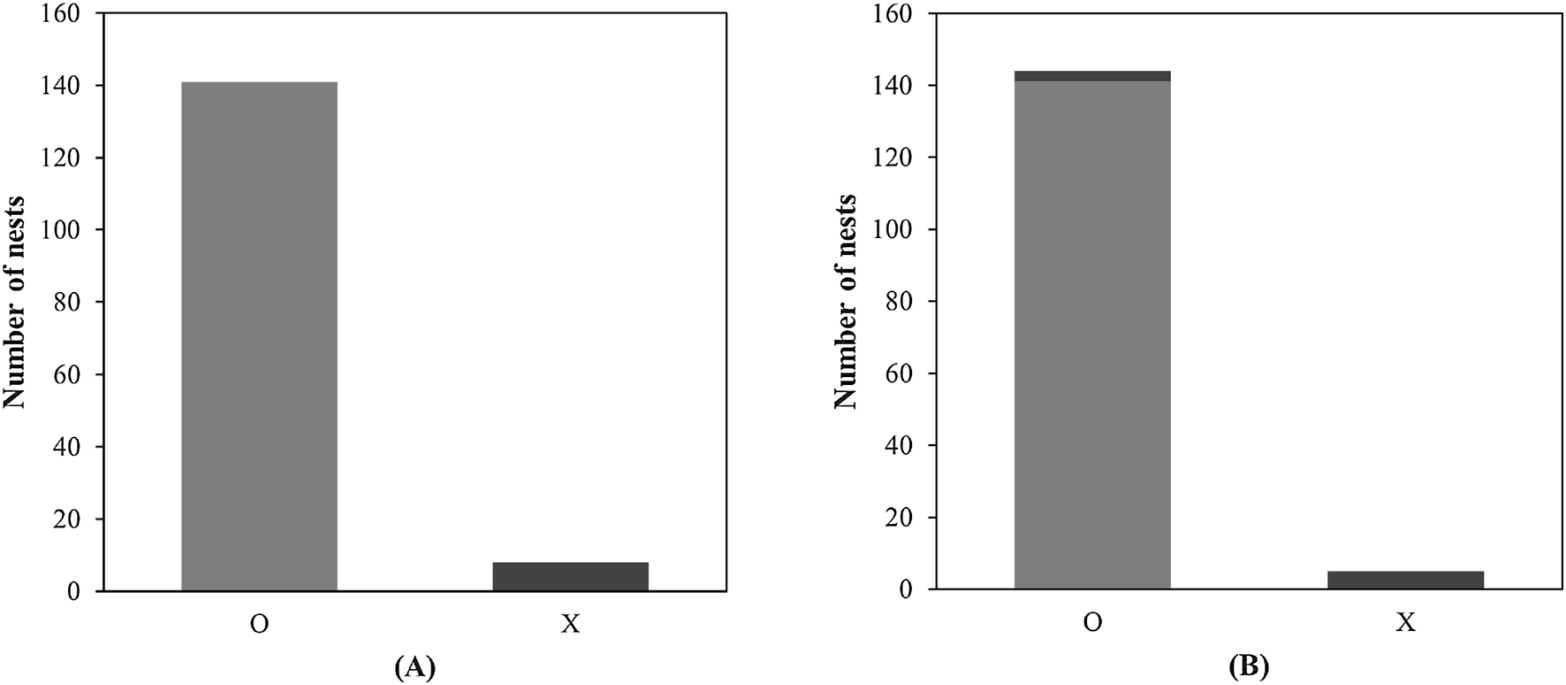
The relationship between human presence and the breeding nests of barn swallows(n = 149). (**A**) illustrates variations in the presence of people in buildings where swallows attempted breeding nests. “O” represents buildings occupied or used by people, and “X” represents vacant buildings. (**B**) is a reclassification of A's results, specifically focusing on nests located in vacant buildings and categorizing them based on the intensity of human activity in the vicinity. “O” signifies human activity, while “X” represents the absence of human activity. According to (**B**), among the 8 nests in vacant buildings from (**A**), 3 nests were actually located where substantial human activity occurs, and a total of 144 nests were situated in places with high human activity.

149 barn swallow nests were researched in 14 villages on three islands, breeding of 141 nests (approximately 94.6%) occurred in 83 buildings where humans reside, and 8 nests (approximately 5.4%) were located in 7 vacant buildings (Fig. 1A). The study site included 684 buildings in which humans reside and 169 empty buildings, according to the results, a higher ratio of nests were observed in buildings where humans reside (χ^2^ test = 23.70, *p* < 0.001).

Because human activities frequently occur even in empty buildings, cases where people visited the building or worked in the building were reclassified as buildings with human activities (Fig. 1B). Including empty buildings with human activity, breeding of 144 nests (approximately 96.6%) occurred in 85 buildings with human activities, and breeding of 5 nests occurred in 5 buildings with no human activities (approximately 3.4%). Barn swallows build new nests or reuse old nests. Of 117 nests, there were 114 nests that reproduced by reuse of nests (approximately 97.4%), which were located in inhabited buildings, and only three nests (approximately 2.6%) were located in empty buildings. Regarding new nests, 27 (84.4%) of 32 nests were located in buildings inhabited by humans, and five nests (15.6%) were located in empty buildings.

### Relationship between the frequency of human activity and the number of barn swallow nests

**Figure 2:**
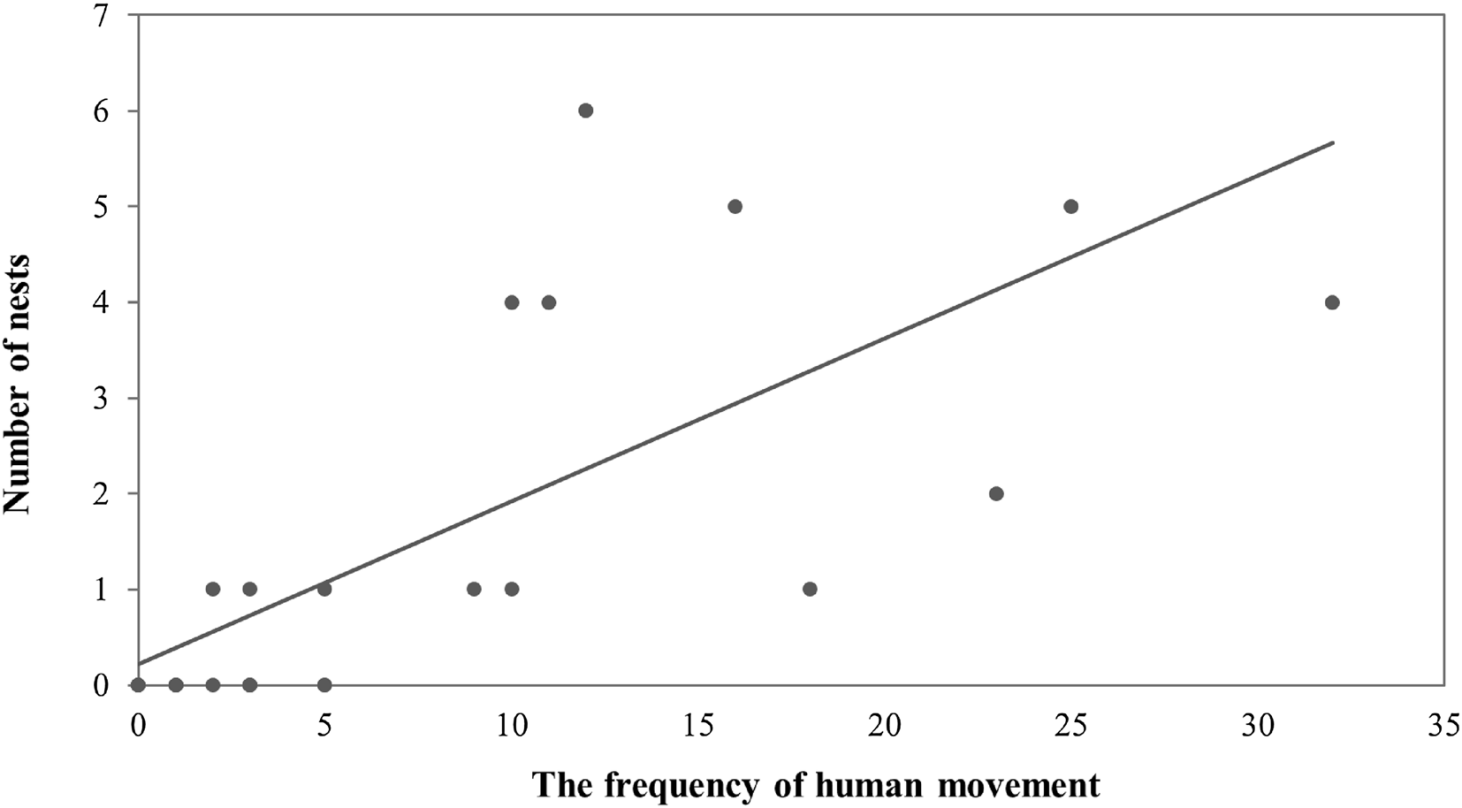
A graph showing the relationship between the frequency of human movement and the number of barn swallow nests. The result of Poisson analysis indicated that the more human movement, the greater the tendency of increasing the number of swallow nests (Poisson regression, *p* < 0.0001).

The result of analysis of the relationship between the frequency of human movement and the number of barn swallow breeding nests performed at the time of breeding place selection s demonstrated that the number of swallow nests increased as the frequency of human movement increased (Fig. 2, Poisson,* p* < 0.0001). Almost no barn swallow nests were found on the roadside where the frequency of human movement was less than 5 during the 2 h of study, however, up to six nests were observed where the frequency of movement was 10 or more.

### Characteristics of the building and walls where the swallow's nest was located

**Figure 3:**
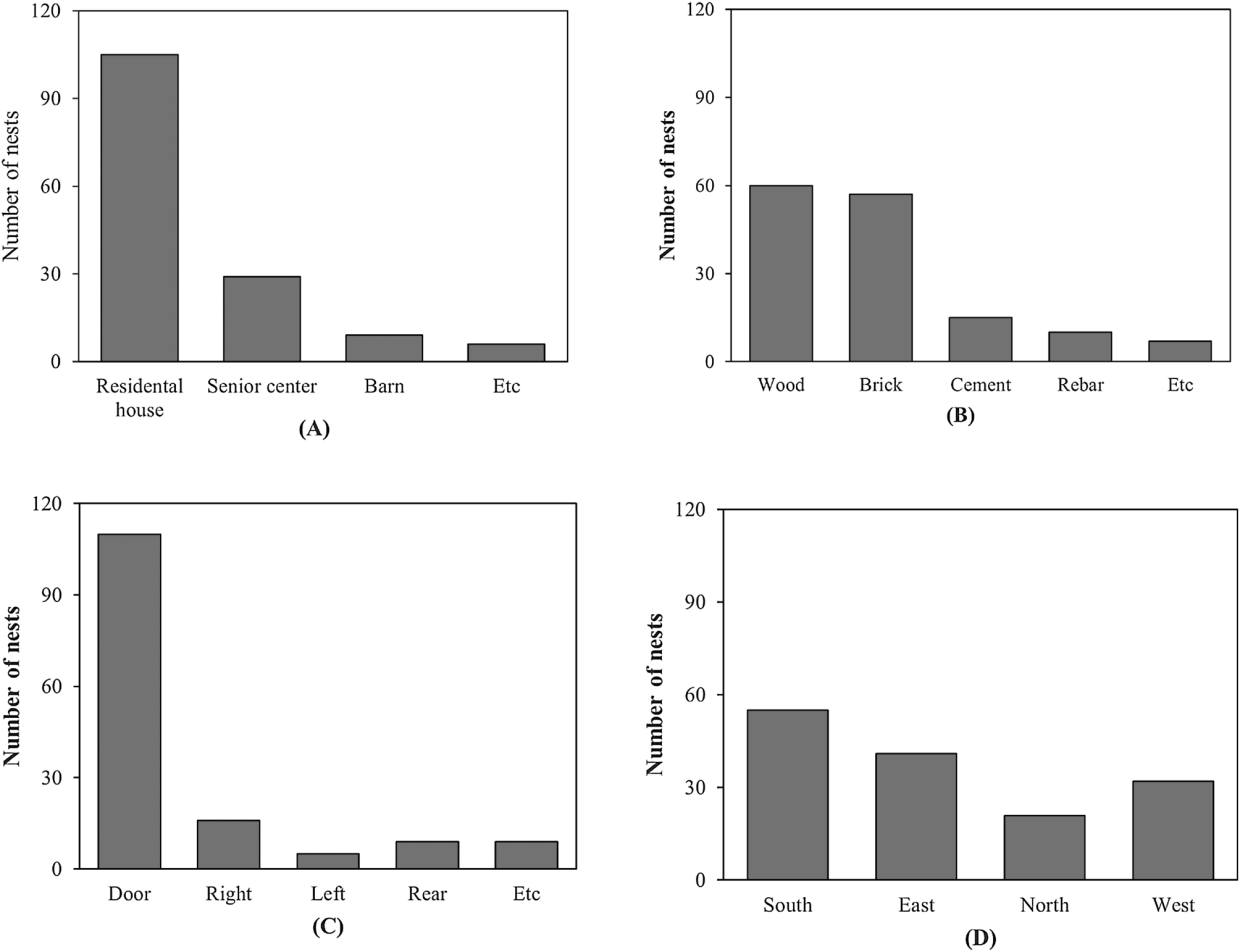
Comparison of the number of barn swallow nests according to the building characteristics. (**A**) is showing the number of nests according to building type. "Etc" refers to places that are difficult to define as a temple or building. (**B**) is showing the number of nests according to wall materials. "Etc" refers to nests located in places that are difficult to define based on materials such as lamps, clotheslines. (**C**) is the number of nests corresponding to the side direction of the house where the nests are located. "Etc" includes elements such as front doors, ceiling. (**D**) Graph showing the number of nests according to orientation.

An analysis of the characteristics of the building where the nest was located was performed. Of the 149 nests where breeding was attempted, 105 nests (70.5%) were located in a residential house, and 29 nests (19.5%) were located in a senior center (Fig. 3A). Nine nests (6.0%) were located in a barn, and six nests (4.0%) were located in other areas. Regarding the material of the wall to which the nests were attached, 60 nests (40.3%) located on wood, 57 nests (38.3%) located on bricks, 15 nests (10.1%) located on cement, 10 nests (6.7%) located on rebar, and seven nests on other materials (4.8%) were examined (Fig. 3B). Considering the attached side (Fig. 3C), 110 nests (73.8%) were located on the side of the door, 16 nests (10.7%) were located on the right side of the door, five nests (3.4%) were located on the left side of the door, and nine nests were located on the other side of the door (6.0%). According to the four cardinal points of the wall where the barn swallow’s nest was located (Fig. 3D), 41 nests (27.5%) were located in the east, 32 nests (21.5%) were located in the west, 55 nests (36.9%) were located in the south, and 21 nests (14.1%) were located in the north.

### The relationship between the distance from resources required for reproduction and the number of barn swallow nests

The results of analysis of the relationship between the number of nests and the area where resources are required for breeding and habitation of barn swallows showed no significant tendency with regard to the distance to water, rice fields, mountains in each study site (Table 1, linear regression analysis). However, relationship of buildings can infer that there is a tendency indeed.

**Table 1:** Association analysis of barn swallow nest counts and resources through linear regression analysis.

Resource	B	SE	*β*	*t*	*p-value*
Intercept	7.547	7.328		− 1.030	0.330
Water	0.15	0.014	− 0.19	− 0.623	0.547
Field	0.067	0.033	0.514	1.846	0.095
Mountain	− 0.111	0.064	0.087	0.271	0.792
Building	0.486	0.214	0.977	2.253	0.051

### The breeding ecology of swallows according to human habitation

**Figure 4:**
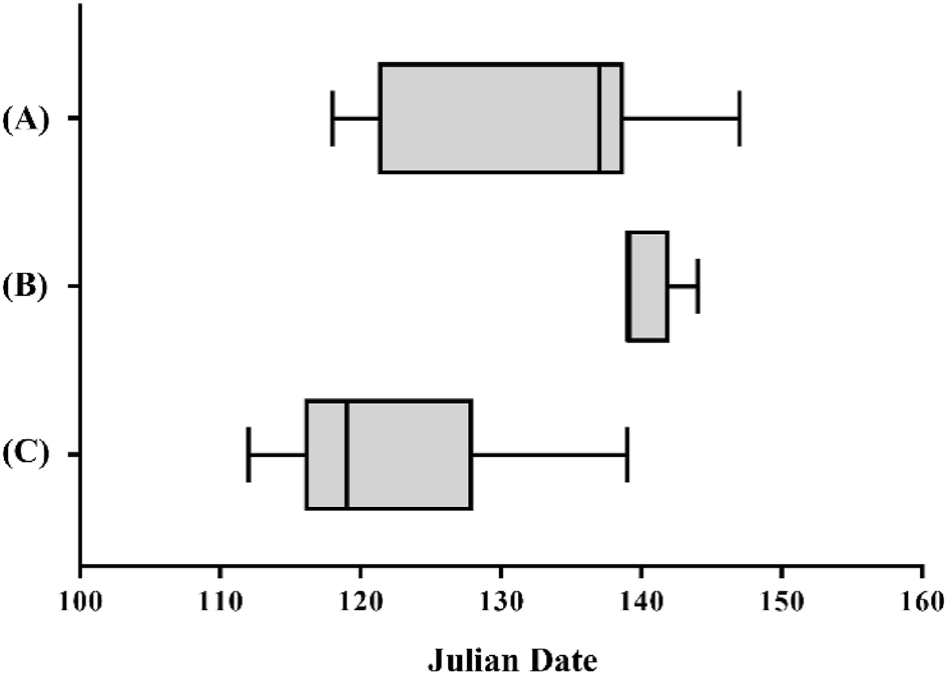
A graph comparing first laying date depending on whether there were humans in the building and whether the nest was reused or newly built. (**A**) represents the case where birds reuse nests originally found in buildings used by humans, with a total of 86 nests located. (**B**) represents the scenario where birds reuse nests in vacant houses without human presence, with 6 nests located. (**C**) represents the situation where birds build new nests for breeding in buildings used by humans, with a total of 12 nests analyzed.

A total of 104 nests for which the laying date could be accurately determined were divided into groups according to whether or not humans resided in that location and whether the nests were reused or new, and the laying date of each group was compared. The number of samples was small and obtaining an accurate estimate of the first laying date was difficult; therefore, new nests located in empty buildings were excluded from the analysis. In an analysis using a nonparametric method (Kruskal–Wallis test), the results for the average first days of laying new nests in buildings inhabited by humans, nests reused in buildings inhabited by humans, and nests reused in empty buildings were significant (Fig. 4, *p* < 0.001), The result of a post-hoc test (Bonferroni) indicated that the earliest laying date occurred with a reused nest in a building with humans. The result of a post-hoc test (Bonferroni) indicated that the earliest first laying dates occurred in reused nests located in buildings with humans.

There was no significant difference in the rate of breeding success according to the type of building, the wall material where the nest was located, and the side where the nest was located. However, among the 149 nests in which all breeding was attempted, the nests were classified according to those located on a wall with a significant level of human activity (127 nests) and those that were not (22 nests), and a comparison of the reproductive ecology was performed. The result of a comparison of the number of eggs with the number of offspring that succeeded in fledging indicated that the number of eggs did not satisfy the significance level of 5%, however, a high average number of eggs was observed in the nest installed on the wall with human activity. In contrast, a significantly higher number of offspring that succeeded in fledging was observed in nests located on walls where humans were active compared with nests that were not. The rate of reproductive success was also significantly higher in nests built on walls with human activity (Table 2).

**Table 2:** Comparative analysis of reproductive differences (egg, offspring, reproductive success rate) in nests according to human activity.

	Eggs	Offsprings	Reproductive success rate (%)
(A)	4.01	3.54	75.00
(B)	3.18	1.95	43.64
*p-value*	0.092	< 0.0001	< 0.0001

(A) represents the analysis of nests located on wall surfaces where human activity is prominently observed and (B) represents the analysis of nests situated on wall surfaces with minimal human activity. Significant differences in the number of offspring and breeding success rates can be observed in nests located in areas with high human activity.

## Discussion

In this study, barn swallow breeding was found to occur within close proximity to human dwellings, and the significant influence of humans on the reproduction of barn swallows has been verified. In comparison of human occupancy with the number of breeding nests, attempts at breeding were more likely to occur in buildings inhabited by humans during the breeding season. Even when a building was empty, human activity was observed in some cases, so that cases involving visible human activity and those not involving human activity were classified as an empty building; a significantly higher number of nests were located in buildings with human activity. Other factors that might affect selection of the nest location (building type, orientation, and wall material where the nest is located) were not regarded as having a significant effect on the selected location of the barn swallow nest. Swallow nests were located mainly in houses and senior centers, buildings with significant human activity. Most of the walls where the nest was located appeared to be facing south. Some birds choose a southern nest entrance orientation in order to gain the advantage of maintaining the temperature of the nest^18^, however, there are differences among species^19,20^. In addition, according to the results of this study, approximately 96.6% of the barn swallow nests were located under the structure that serves as the eaves; due to its structure, sunlight does not reach either the inside or the outside of the nest, thus it is not possible to obtain this advantage. The wall material where the nest is located is not significantly different from wood, brick, and cement, therefore, it is presumed that it has no significant effect on selection of the nest location. This fact is supported by the findings of studies^21–23^ that indicated no preference for directionality in nest selection of some birds. In this study, no significant relationship was observed between the number of barn swallow nests and the distance from potential resources available to barn swallows, whereas the result from comparison of the relationship with the number of nests according to the frequency of human activity showed a significant relationship. In light of these findings, it can be inferred that the presence or absence of humans exerts a more pronounced influence on the choice of a breeding site for barn swallows when compared to other factors. The following possibilities can be inferred from the current result showing that barn swallow nesting occurs in buildings where humans are active and in areas with significant human movement. It is hypothesized that building a nest near humans provides protection from predators and the rate of breeding success is high, with active selection of a location with significant human activity. Moller^24^ reported that the nest predation rate of barn swallows nesting indoors was lower than the nest predation rate nesting outdoors. Therefore, the conclusions drawn from the correlation between human activity and nest count suggest a potential association with the presence of predators.

Predators have a profound effect on bird reproduction^14,25^. Not only do they feed on eggs and chicks, but the presence of predators also limits the parental activity required for reproduction. In fact, many studies have reported on changes in prey species caused by predators^26–28^. In addition, it is often observed in nature that various birds protect their nests by establishing species of predators as neighbors who may pose a threat to their potential predators^29^. For example, there is variation in the number of nests of the azure-winged magpie (*Cyanopica cyanus*) depending on the rate at which Japanese lesser sparrowhawks (*Accipiter gularis*) expel their natural enemies^30^. Another example is that of great snow geese (*Chen caerulescens atlantica*), who consider larid and snowy owls (*Bubo scandiacus*) as protector species that will protect their nests and therefore breed near them^31^. When choosing their breeding grounds, barn swallows build their nests in close proximity to humans because they will provide protection from predators such as snakes, rodents, owls, bats, and raptors^24,32^. When breeding was attempted, there was no significant difference in the number of eggs in nests with and without human activity, however, a significant difference was observed between the number of offspring and the rate of reproductive success. This means that where there is a high level of human activity, there is better hatching of offspring as well as more successful fledging of offspring. Nesting within the range of human activity would limit the activity of potential predators of barn swallows, which would reduce the effect of predators on the brooder activity of barn swallows, thereby increasing the rate of reproductive success^24^. In this case, it is possible that barn swallows were using humans as a means of avoiding predators.

Barn swallows not only build their nests near a door, through which humans often enter and exit, but also in locations that are within reach of humans. This probably occurs because barn swallows do not perceive humans as a threat to their nests. Barn swallows have traditionally been animals that have a close relation to humans. The barn swallow has a positive association in many European countries; it is a herald of spring and is familiar enough to make a frequent appearance in beliefs and superstitions of the past^13^. They stayed from March until September and believed that they would observe human behavior and report back to the gods. Therefore, according to a shamanic belief, swallows are objects of worship and if they overdo it, the family will be cursed with bad luck. These folk beliefs have faded, however, there is still a belief among local elders that barn swallows should be protected. In the case of our study sites, elderly people aged 65 and over account for 42% of the population, so that the sentiment toward protection of barn swallows is still high in the surveyed area. In addition, it is a representative animal associated with emotion that has been interacting with people for a long time due to its homing instinct^9,12^ which memorizes the nest where it lived, so that it returns in spring.

The swallow (Hirundinidae*)* nest has various forms, including those of the studied species, Barn swallow^33^. Tree swallow (*Tachycineta bicolor*) builds the nests using moss and grass, and the Bank swallow (*Riparia riparia*) makes holes in soil walls, building its nest with twigs and weeds. The cliff swallow (*Petrochelidon pyrrhonota*) builds its nest in the shape of a gourd with a narrow mouth in dry mud on the side of a cliff. Although they belong to the same family of swallows, nest material and shape vary according to species, thus they are capable of rapid adaptation to a specific breeding environment. Based on characteristics such as selectively building nests in areas with active human presence and exhibiting positive effects on reproduction in places influenced by humans, it is inferred that the barn swallow has also shown rapid adaptation.

## Data Availability

The dataset is available from the corresponding authors upon request.
